# The effects of peritoneal dialysis on QT interval in ESRD patients

**DOI:** 10.1186/s12882-022-02685-y

**Published:** 2022-02-18

**Authors:** Wenjing Zhang, Yu Liang, Jia Lv, Yan Li, Jiping Sun

**Affiliations:** Department of Nephrology, The First Affiliated Hospital of Xi’an Jiaotong University, 277 West Yanta Road, Xi’an, 710061 Shannxi China

**Keywords:** Peritoneal Dialysis, QT interval, Corrected QT interval (QTc)

## Abstract

**Background:**

Patients with chronic kidney disease (CKD) are at a high risk of fatal arrhythmias. The extended corrected QT (QTc) interval is a hallmark of ventricular arrhythmias and sudden cardiac death. Previous studies have shown that QT interval and QTc are prolonged with the decline in renal function. However, there were no available results for patients with peritoneal dialysis (PD). In this study, we examined changes in QT interval and QTc in patients with end-stage renal disease (ESRD) who underwent peritoneal dialysis.

**Methods:**

A total of 66 ESRD patients who received PD, including 50 males and 16 females, with an average age of 43.56 ± 15.15 years, were enrolled. The follow-up lasted 1 year. The demographics and the etiology of patients were recorded. QTc and clinical/biochemical indexes before dialysis and at 6 and 12 months were determined and analyzed. Dialysis adequacy and peritoneal transport function were assessed in each patient. Analysis of variance (ANOVA), least significant difference (LSD) or Tamhane’s T2, Paired T-test, Chi-square test, multiple linear regression analysis, and Pearson correlation coefficient were used to analyze the data. *P* < 0.05 was considered as statistically significant.

**Results:**

With reference to etiology, 37 patients (56.06%) had chronic nephritis, and 11 (16.67%) had diabetic nephropathy. Most of the peritoneal transport functions were low average transport (25, 37.88%), while the least were high transport (2, 3.03%).During the follow-up period, all patients had adequate peritoneal dialysis. Compared with a baseline before dialysis, anemia, low albumin, blood pressure, blood urea nitrogen, creatinine, uric acid, potassium, calcium, phosphorus, and parathyroid hormone improved after 6 and 12 months, while the residual renal function gradually decreased during the follow-up. The mean QTc of all patients was stable during the follow-up period. According to gender, the QTc in males and female patients were similar. Before PD, diastolic blood pressure, calcium concentration, and hemoglobin level were negatively correlated with QTc in end-stage renal disease patients; After PD, the observed clinical indexes were no longer relevant to QTc.

**Conclusion:**

Unlike hemodialysis-induced QTc prolongation, PD did not increase the patient’s QT interval and QTc interval, which suggested that myocardial electrical activity might be more stable in patients with adequate peritoneal dialysis.

## Background

Chronic kidney disease (CKD) is a global public health problem with an estimated prevalence of 11-13% [1], and one of the leading causes of death. CKD patients have an increased risk of developing life-threatening cardiovascular complications, including ventricular arrhythmias and sudden cardiac death. For example, cardiovascular disease (CVD) mortality is 30 times higher in patients undergoing dialysis than in the general population [2]. According to the USA National Institutes of Health, National Institute of Diabetes and Digestive and Kidney Disease, in 2020, approximately 55.2% of patients undergoing dialysis were associated with CVD, and 44.2% with cardiac arrest or arrhythmia [3].

QT interval represents the duration of ventricular depolarization and repolarization, and it is closely related to heart rate. The QT interval shortens with accelerating heart rate in normal people. The heart rate is used for correcting the QT interval: corrected QT interval [QTc, QTc = QT/(RR interval in seconds)] [1]. A prolonged QTc interval is defined as QTc greater than 440 ms in males and QTc greater than 460 ms in females [4]. Prolonged QTc interval is found in 12.9% of the general population and in 20.5% of CKD patients [5]. Also, the QTc interval increases by an average of 2.9 ms for each milligram increase in serum creatinine [6]. Current studies have shown that the prolongation of QT interval and QTc may predict malignant ventricular arrhythmia and sudden cardiac death [7, 8]. Moreover, studies [9–11] have also proved that QT interval and QTc are prolonged with the declination of renal function, especially in patients with maintenance hemodialysis, showing longer QT interval and QTc after dialysis than before dialysis. Yet, it remains unclear how QT interval and QTc change in patients undergoing maintenance peritoneal dialysis (PD). To the best of our knowledge, this study analyzed the changes of QT interval and QTc in PD patients for the first time.

## Methods

### Study subjects

This single-center, the retrospective study enrolled patients with PD treated at the first affiliated hospital of Xi’an Jiaotong University between January 1, 2018 and May 31, 2019. The inclusion criteria were: (1) age > 18 years old; (2) the cause of the disease was not limited. The exclusion criteria were: acute exacerbation of CKD; acute kidney injury; contraindications to peritoneal dialysis and incomplete data; died within one year; varied basic cardiac diseases, such as myocardial infarction, myocardial ischemia, cardiomyopathy, and cardiac dysfunction; varied arrhythmias, such as atrial, junction and ventricular arrhythmias, various conduction blocks, and pacemaker implantation; patients using drugs to prolong QT interval. All eligible patients had no indication of emergency dialysis.

The study was conducted in accordance with the principles of the Declaration of Helsinki, and the ethics committee approved the study protocol of The First Affiliated Hospital of Xi’an Jiaotong University. Because of the retrospective nature of the study, patient consent for inclusion was waived.

### Peritoneal dialysis scheme

All patients underwent manual PD on the day of PD catheter insertion: 1.5% peritoneal fluid, 1000 ml was injected each time and maintained for 1 h; the total dialysis dose was 6000 ~ 8000 ml/d. After 10 days, the dose per time was changed to 2000 ml and maintained for 4 h. The total dialysis dose was 6000 ~ 8000 ml/d. After 1 month, a peritoneal equilibration test and PD adequacy were performed. According to the D/*P* value, the peritoneal transport function was divided into four types: low transport, low average transport, high average transport, and high transport. Adequacy was calculated using Kt/V and Ccr (renal, peritoneal, and total). The PD prescriptions were adjusted according to the above results. After PD for 6 months and 1 year, the dialysis adequacy evaluations were performed again.

### Electrocardiograph

The twelve-lead surface electrocardiogram (ECG) was performed at a speed of 25 mm/s and a voltage of 10 mm/mv before dialysis, and after 6 and 12 months. ECG parameters included heart rate, QT interval, and QTc. The QT interval was measured in all leads of ECG, and the longest QT interval was accepted. The start of the QT interval was measured from the beginning of the QRS complex; the end of the QT interval was measured at the return of the TP baseline or when a U wave was present at the nadir between the T wave and the U wave. The QT interval was measured by an investigator for each patient and was corrected according to heart rate using Bazett’s formula: *QTc = QT/√RR (ms)*, where QTc was the corrected value of the QT interval.

### Fellow up and clinical/biochemical indexes

The follow-up time was 1 year. The demographics, clinical, biochemical indexes, and ECG parameters before dialysis were recorded. After 1 month, the peritoneal transport function was recorded. The clinical biochemical indexes (including blood pressure, hemoglobin, blood urea nitrogen, serum creatinine, uric acid, albumin, potassium, magnesium, sodium, chlorine, calcium, phosphorus, parathyroid hormone, carbon dioxide binding rate, and residual renal function), dialysis adequacy, ECG parameters (including heart rate, QT interval, and QTc) were performed at 6-months and 1 year. The residual renal function (RRF) was calculated using the combined 24-h urinary urea and creatinine clearance.

### Statistical method

The data were presented as means± standard deviation for continuous variables. Analysis of variance (ANOVA) was used to test the difference in the mean sample during the follow-up time. The least significant difference (LSD) or Tamhane’s T2 was further compared whether there was any significant difference between the two groups. Paired T-test was used between the two groups. Comparison of counting data was achieved by using the Chi-square test. Multiple linear regression analysis and Pearson correlation coefficient were used to recognize elements related to QTc interval. *P* < 0.05 was considered to be statistically significant. Analyses were performed by employing SPSS (version 22) statistical software package.

## Results

### Demographics and clinical and biochemical indexes before dialysis

**Fig. 1:**
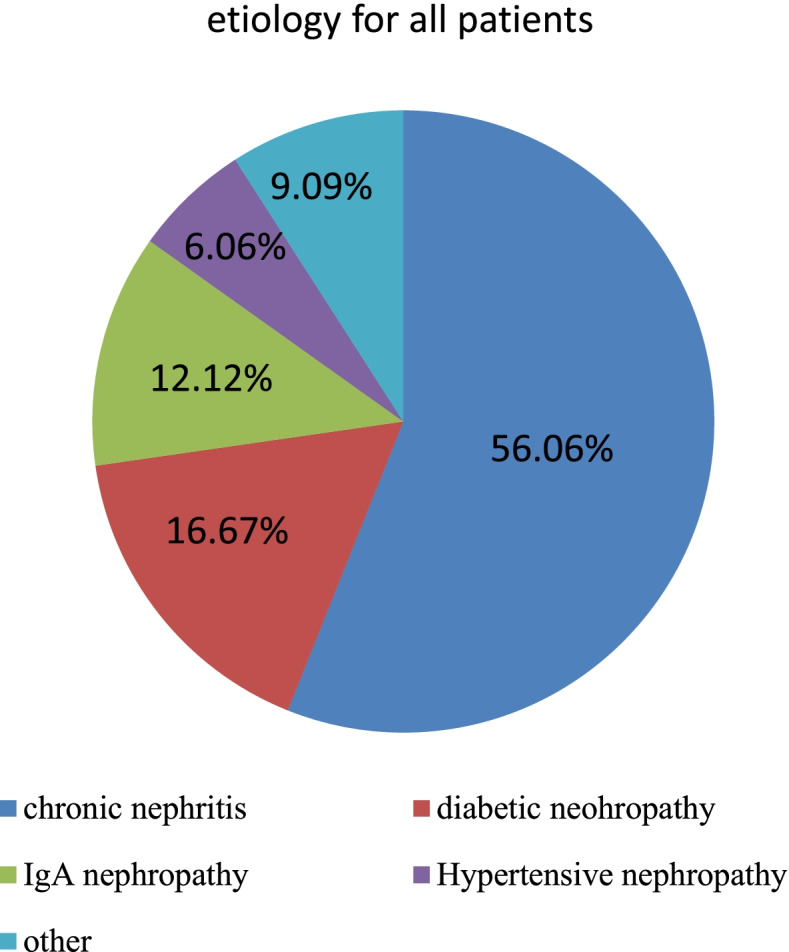
It showed the etiology for all patients. 37 patients (56.06%) had chronic nephritis, 11 patients (16.67%) had diabetic nephropathy, 8 patients (12.12%) had IgA nephropathy, and 4 patients (6.06%) had hypertensive nephropathy

**Fig. 2:**
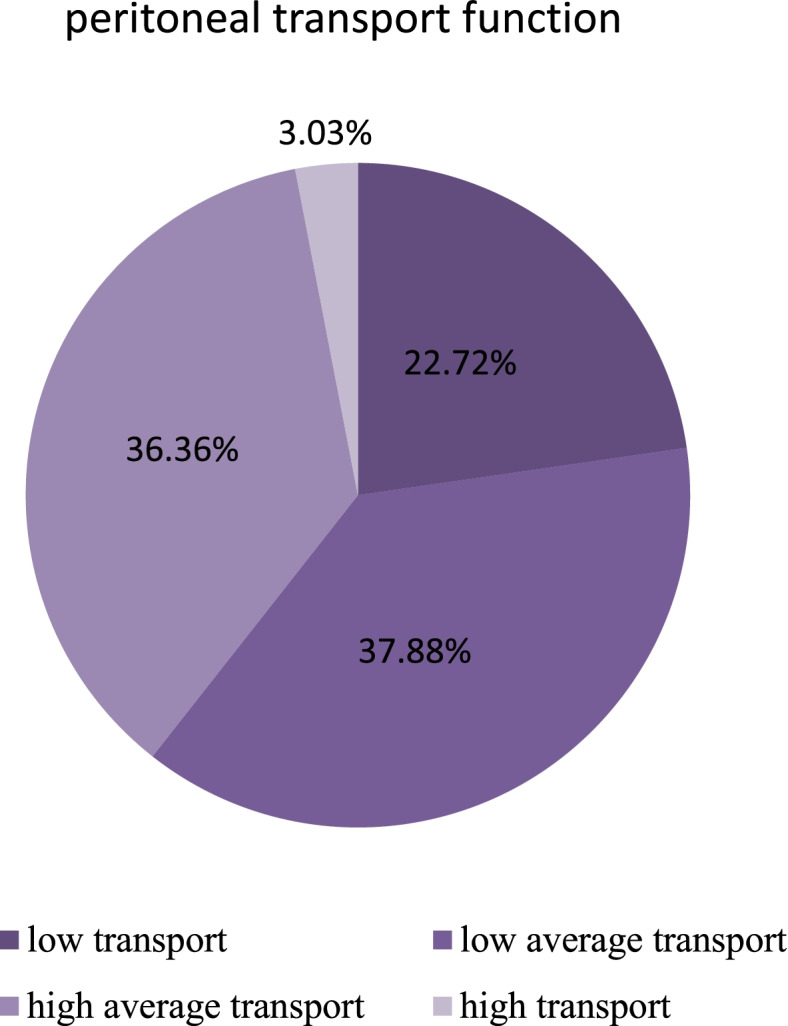
It showed the peritoneal transport function for all patients. 25 patients (37.88%) had low average transport function, 24 patients (36.36%) had high average transport function, 15 patients (22.72%) had low transport function, and 2 patients (3.03%) had high transport function

A total of 66 PD patients, including 50 males and 16 females, with an average age of 43.56 ± 15.15 years (males: 43.74 ± 15.53 years; females: 43.00 ± 15.92 years) were enrolled in the study. In terms of etiology, 37 patients (56.06%) had chronic nephritis, 11 patients (16.67%) had diabetic nephropathy, and 8 patients (12.12%) had IgA nephropathy (Fig. 1). The peritoneal transport test showed that 25 patients (37.88%) had low average transport, 24 patients (36.36%) had high average transport, 15 patients (22.72%) had low transport, and 2 patients (3.03%) had high transport (Fig. 2).

### Clinical and biochemical indexes during the follow-up period

Systolic blood pressure, diastolic blood pressure, albumin, blood urea nitrogen, creatinine, uric acid, potassium, magnesium, chlorine, phosphorus, calcium, hemoglobin, and carbon dioxide binding rate after 6 months and 1 year were better than those before dialysis, while the blood sodium level was higher and the eGFR (estimated glomerular filtration rate) was lower (all *P* < 0.05) (Table 1a, b, Table 2a, b); all clinical and biochemical indexes were not statistically significant between 6 months and 12 months (*P* > 0.05), (Table 2 a, b).

**Table 1:** Comparison of clinical biochemical indexes during follow-up period (Mean ± SD)

a								
Items	Systolic blood pressure (mmHg)	Diastolic blood pressure (mmHg)	Albumin (g/L)	Blood urea nitrogen (mmol/L)	Creatinine (umol/L)	Uric acid (umol/L)	Potassium (mmol/L)	Magnesium (mmol/L)
pre-PD	148.64 ± 20.13	89.29 ± 15.05	33.82 ± 5.11	31.27 ± 12.38	925.25 ± 343.76	515.55 ± 144.03	4.59 ± 0.83	1.14 ± 0.24
6 months	131.71 ± 20.14	81.93 ± 12.69	38.03 ± 5.16	17.36 ± 4.14	774.30 ± 274.32	386.45 ± 63.70	4.19 ± 0.66	0.99 ± 0.14
1 year	134.77 ± 18.58	84.02 ± 12.06	37.07 ± 4.80	18.98 ± 5.66	888.60 ± 319.12	378.08 ± 75.63	4.11 ± 0.65	0.99 ± 0.13
F value	13.083	4.953	11.972	49.587	3.758	33.843	7.580	13.960
*P*	0.000	0.008	0.000	0.000	0.025	0.000	0.001	0.000
b								
Items	Sodium (mmol/L)	Chlorine (mmol/L)	Phosphorus (mmol/L)	Calcium (mmol/L)	Parathyroid hormone (pg/ml)	Hemoglobin (g/L)	Carbon dioxide binding rate (mmol/L)	eGFR (ml/min)
pre-PD	138.82 ± 4.44	100.38 ± 5.59	2.11 ± 0.60	1.88 ± 0.30	314.65 ± 208.71	83.48 ± 18.12	18.42 ± 3.92	5.76 ± 2.17
6 months	141.88 ± 3.12	98.44 ± 3.50	1.35 ± 0.38	2.13 ± 0.23	327.63 ± 219.86	103.45 ± 20.77	22.95 ± 3.20	3.77 ± 2.70
1 year	141.39 ± 3.25	98.17 ± 3.60	1.49 ± 0.44	2.15 ± 0.17	362.42 ± 240.12	101.02 ± 18.12	23.72 ± 5.05	3.63 ± 5.23
F value	12.207	4.476	42.104	22.597	0.630	20.315	30.097	7.093
*P*	0.000	0.013	0.000	0.000	0.534	0.000	0.000	0.001

**Table 2:** Comparison of clinical biochemical indexes between Groups during Follow-up Period

a									
Items		Systolic blood pressure	Diastolic blood pressure	Albumin	Blood urea nitrogen	Creatinine	Uric acid	Potassium	Magnesium
*P* value	Pre-PD VS 6 months	0.000	0.003	0.000	0.000	0.008	0.000	0.003	0.000
	Pre-PD VS 1 year	0.000	0.036	0.001	0.000	0.513	0.000	0.001	0.000
	6 months VS 1 year	0.417	0.417	0.320	0.251	0.059	0.899	0.583	1.000
b									
Items		Sodium	Chlorine	Phosphorus	Calcium	Parathyroid hormone	Hemoglobin	Carbon dioxide binding rate	eGFR
*P* value	Pre-PD VS 6 months	0.000	0.062	0.000	0.000	0.747	0.000	0.000	0.000
	Pre-PD VS 1 year	0.001	0.033	0.000	0.000	0.269	0.000	0.000	0.025
	6 months VS 1 year	0.811	0.970	0.227	0.901	0.436	0.507	0.731	0.997

### Dialysis adequacy during the follow-up period

In terms of dialysis adequacy, the total Kt/v were greater than 1.7(at 6 months: 2.12 ± 0.61; at 1 year: 2.19 ± 0.90; *P* = 0.648), and the total Ccr were higher than 50 L (at 6 months: 92.37 ± 42.71 L; at 1 year: 92.08 ± 71.53 L; *P* = 0.979, Table 3), which indicated that patients had reached the standard of dialysis.

**Table 3:** Dialysis adequacy during the follow-up period (Mean ± SD)

Items	PKt/v	UKt/v	TKt/v	PCcr	UCcr	TCcr
6 months	1.43 ± 0.39	0.67 ± 0.51	2.12 ± 0.61	40.48 ± 8.60	51.90 ± 43.17	92.37 ± 42.71
1 year	1.50 ± 0.41	0.69 ± 1.08	2.19 ± 0.90	43.34 ± 10.30	48.74 ± 77.38	92.08 ± 71.53
*t* value	−1.187	−0.205	−0.819	−1.049	0.587	0.058
*P*	0.240	0.838	0.416	0.300	0.560	0.954

Note: *PKt/v* peritoneal urea clearance index; *UKt/v* renal urea clearance index; *TKt/v* total urea removal index; *PCcr* peritoneal creatinine clearance rate; *UCcr* renal creatinine clearance rate; *TCcr* total creatinine clearance rate

### Electrocardiograph parameters during the follow-up period

During the whole follow-up period, the heart rate, QT interval, and QTc of all patients were similar, showing no statistically significant differences (*P* > 0.05 respectively, Table 4 and Table 5). According to gender, the QTc in males (*P* = 0.363) and female patients (*P* = 0.206) were similar (Table 6 and Table 5). Moreover, we found that the percentage of prolonged QTc in the population before and after PD were similar (*P* > 0.05, Table 7).

**Table 4:** Comparison of ECG parameters during the follow-up period (Mean ± SD)

Items	Heart rate (bpm)	QT interval (ms)	QTc (ms)
pre-PD	81.90 ± 12.69	378.54 ± 39.60	413.49 ± 29.95
6 months	79.55 ± 10.51	390.57 ± 54.33	423.05 ± 51.96
1 year	80.12 ± 13.48	375.88 ± 43.57	409.29 ± 32.32
F value	0.534	1.365	1.592
*P*	0.587	0.258	0.207

Note: *QTc* corrected QT interval

**Table 5:** Comparison of ECG indexes between groups during follow-up period

Items		Heart rate			QT interval			QTc		
		Total	Male	Female	Total	Male	Female	Total	Male	Female
*P* value	Pre-PD VS 6 months	0.343	0.386	0.776	0.184	0.166	0.928	0.206	0.212	0.788
	Pre-PD VS 1 year	0.443	0.252	0.481	0.754	0.794	0.137	0.553	0.946	0.128
	6 months VS 1 year	0.826	0.836	0.382	0.120	0.267	0.167	0.081	0.203	0.117

Note: *QTc* corrected QT interval

**Table 6:** Comparison of ECG parameters in different genders during the follow-up period (Mean ± SD)

Items	Heart rate (bpm)		QT interval (ms)		QTc (ms)	
	Male	Female	Male	Female	Male	Female
pre-PD	80.91 ± 12.86	84.81 ± 12.11	378.68 ± 40.68	378.13 ± 37.54	412.49 ± 30.87	416.44 ± 27.79
6 months	78.52 ± 10.91	83.33 ± 8.31	393.58 ± 60.15	379.56 ± 21.97	424.00 ± 57.86	419.56 ± 20.65
1 year	77.93 ± 12.27	88.27 ± 15.25	381.32 ± 41.61	355.64 ± 46.74	411.90 ± 32.29	399.55 ± 31.99
F value	0.744	0.437	1.047	1.428	1.023	1.658
*P*	0.477	0.650	0.354	0.254	0.363	0.206

Note: *QTc* corrected QT interval

**Table 7:** Comparison of the percentage of prolonged QTc in different genders during follow-up period (%)

Items	Male	Female
Pre-PD	18%(9/50)	0%(0/16)
6 months	22%(11/50)	0%(0/16)
1 year	20%(10/50)	6.25%(1/16)
χ^2^ value	0.250	2.043
*P*	0.882	0.360

### Multiple linear regression analysis and Pearson correlation coefficient

We evaluated SBP, DBP, ALB, eGFR, BUN, Scr, UA, potassium, magnesium, sodium, chlorine, phosphorus, calcium, PTH, hemoglobin, and carbon dioxide binding rate. And the “Enter” mode was used to determine which indexes were meaningful. Before PD, multiple linear regression analysis and Pearson correlation coefficient analysis showed that QTc was correlated with diastolic blood pressure, calcium, and hemoglobin, and the regression coefficients were − 0.870, − 42.434, and − 0.483, respectively (Table 8). Nevertheless, when the patients started PD, the observed clinical indexes no longer affected QTc.

**Table 8:** Influencing factors significantly correlated with QTc prolongation in ESRD (pre-PD)

Characteristics	correlation coefficient (r)	*P* values	Regression coefficient	95% confidence intervals	T values	*P* values
diastolic blood pressure	−0.261	0.039	−0.870	−1.508 to 0.232	−2.745	0.009
calcium concentration	− 0.360	0.004	−42.434	−71.680 to − 13.187	−2.920	0.005
hemoglobin	−0.432	0.000	−0.483	− 0.943 to 0.022	−2.108	0.041

## Discussion

CKD is an increasing public health problem that can lead to coronary events [12] and reduce glomerular filtration rate (GFR), which is an independent risk factor of cardiovascular mortality caused by acute myocardial infarction, heart failure, thromboembolic disease, and sudden cardiac death (SCD). These events account for 26.5% of all-cause mortality and 64% of cardiac mortality in ESRD patients [13, 14].

QT interval represents the total time course of ventricular depolarization and repolarization. It varies with heart rate. The extended QTc interval is a hallmark of ventricular arrhythmias, SCD, and all-cause mortality [15]. Previous studies [9–11] have proved that with the declination of renal function, the QT interval and QTc are prolonged, especially in maintenance hemodialysis patients. Yet, changes in QT interval and QTc change in patients undergoing maintenance PD have not been explored.

In this study, we examined changes in QT interval and QTc in 66 ESRD patients who underwent peritoneal dialysis. Firstly, we observed the therapeutic effect on the patients. During the whole follow-up period, and especially after 6 and 12 months post dialysis, the dialysis adequacy was up to standard with a total Kt/v > 1.7, and a total Ccr > 50 L.Furthermore, the blood pressure control, anemia correction, malnutrition, calcium, phosphorus metabolism disorders, and acid-base balance disorders of patients showed significant improvement during PD compared to those before dialysis. At the same time, after 6 and 12 months, all clinical indexes were similar, and the differences were not statistically significant. Secondly, the changes of QT interval and QTc were observed in PD patients during dialysis. Liu et al [9] reported that the prolongation of QTc interval worsened with the decreased in renal function. Among patients with CKD stages 3, 4, 5 and patients who underwent hemodialysis, the proportion of patients with prolonged QTc and severely prolonged QTc were 32.43 and 1.4%, 40.23 and 6.9%, 59.06 and 10.1%, 64.31 and 12.6%, respectively [9]. Covic et al [10] and Malhis et al [11] observed an increase in QT interval and QTc in the pre- and post-hemodialysis period. In this study, however, QT interval and QTc were not prolonged when patients underwent PD treatment. Due to different gender, the definition of QT interval and QTc prolongation remained different. Therefore, the changes of QT interval and QTc before and after PD in male and female patients were compared, respectively. The obtained data showed that the QT interval and QTc were not prolonged before and after PD in both males and females and were similar after PD for 6 months and 12 months.

It remains unclear why the QT interval and QTc were significantly longer after hemodialysis but not after PD. The prolongation of QT interval was caused by increased inward current (i.e., the sodium or calcium channels) or a K^+^ decreased outward current (i.e., potassium channel). The currents (Ikr and Iks) have a key role in myocardial repolarization. A prolonged action potential duration (APD) could lead to early changes after depolarization that is caused by inward depolarizing currents (L-type Ca^2+^ channels and Na^+^-Ca^2+^ exchange currents), inducing ventricular arrhythmias like torsade de pointes (TdP). ESRD patients are often accompanied by serious electrolyte acid-base balance disorder, such as hyperkalemia, hypocalcemia, metabolic acidosis, while potassium, calcium, magnesium and metabolic acidosis are important factors for electrical stability of the myocardium [16, 17]. It is well known that hemodialysis can quickly stabilize electrolyte acid-base balance disorder in ESRD patients. The low concentrations of potassium and calcium in the dialysate can be quickly removed potassium in serum. However, a large number of studies have shown a negative correlation between QTc interval change and calcium concentrations and potassium reduction during hemodialysis. Sherif et al [6] found that each mmol/L increase of serum K+ concentration might result in a 16 ms reduction of the QTc interval. Moreover, Alabd et al [18] have reported a negative correlation between decreased serum potassium and the change of QTc duration before and after dialysis. The higher decrease in the serum potassium has been associated with the longer QTc after dialysis. Also, Genovesi and colleagues [19] discovered that compared to patients who used dialysate with higher concentrations of potassium and calcium, patients who used dialysate with lower concentrations of potassium and calcium more had QTc intervals greater than 440 ms.

The non-prolongation of QT interval and QTc in PD patients might be related to the following: firstly, compared with hemodialysis, PD has a lower ability to remove toxins per unit time, so most PD patients usually undergo continuous ambulatory peritoneal dialysis (CAPD). However, this method avoids drastic changes in the concentrations of various ions in the serum in a short time, and the concentration of various ions is in a relatively stable state. Secondly, peritoneal dialysate has two different calcium concentrations: physiological calcium and high calcium. Therefore, the appropriate calcium concentration dialysate is chosen for patients according to the patients’ serum calcium concentrations. Although peritoneal dialysate is potassium-free dialysate, the patients have complete pre-dialysis education. They can be supplemented with potassium tablets and eat foods that are rich in potassium during PD to maintain the serum potassium in a stable state and avoid hypokalemia.

The heart’s blood supply depends on diastolic perfusion, and the impact on myocardial blood supply is more direct if the diastolic blood pressure remains low. This effect is particularly prominent in patients with coronary artery stenosis and left ventricular hypertrophy. Low diastolic blood pressure can cause subendocardial myocardial ischemia that can significantly affect the ventricular repolarization process, resulting in QT and QTc prolongation. Renal anemia is a frequent complication in CKD patients. The prevalence of QT prolongation in patients with anemia is common [20, 21]. The pathophysiological links between anemia and prolonged QT intervals are most probably hypoxia, autonomic dysfunction, and decreased myocardial oxygen supply. In addition, the impairment of delayed rectifier potassium channels and calcium channels might explain the changes in repolarization [22]. In our study, multiple linear regression analysis and Pearson correlation coefficient showed that diastolic blood pressure, calcium concentration and hemoglobin levels were influential factors for QTc prolongation before peritoneal dialysis that were negatively correlated with QTc ESRD patients, which is similar to the previous research results [19–21]. However, with the progression of PD, the symptoms and clinical indexes showed significant improvement, and our results showed that the clinical and biochemical indexes did not affect the QT interval and QTc anymore.

This study has a few limitations. This was a single-center retrospective study with a small sample. A multi-center study with a larger sample size with a longer follow-up is required to study the effects of PD on QT interval.

## Conclusion

To sum up, we concluded that peritoneal dialysis did not increase the patient’s QT interval and QTc. A myocardial electrical activity might be more stable in patients undergoing adequate PD.

## Data Availability

Data are available from the corresponding authors on reasonable request.

## Abbreviations

CKD: Chronic Kidney Disease
PD: Peritoneal Dialysis
Kt/V: Urea clearance index
ECG: Electrocardiogram
ESRD: End-stage Renal Disease
SCD: Sudden Cardiac Death
CAPD: Continuous Ambulatory Peritoneal Dialysis
QTc: Corrected QT interval
CVD: Cardiovascular Disease
Ccr: Creatinine clearance rate
RRF: Residual Renal Function
eGFR: Estimated Glomerular Filtration Rate
TdP: Torsade de Pointes
